# MicroRNAs Involved in Intrinsic Apoptotic Pathway during Cisplatin-Induced Nephrotoxicity: Potential Use of Natural Products against DDP-Induced Apoptosis

**DOI:** 10.3390/biom12091206

**Published:** 2022-08-31

**Authors:** Pía Loren, Yuliannis Lugones, Nicolás Saavedra, Kathleen Saavedra, Isis Páez, Nelia Rodriguez, Patricia Moriel, Luis A. Salazar

**Affiliations:** 1 Center of Molecular Biology and Pharmacogenetics, Scientific and Technological Bioresource Nucleus, Universidad de La Frontera, Temuco 4811230, Chile; 2 Doctoral Programme in Sciences with major in Applied Cellular and Molecular Biology, Universidad de La Frontera, Temuco 4811230, Chile; 3 Faculty of Pharmaceutical Sciences, University of Campinas, Campinas 13083970, SP, Brazil

**Keywords:** apoptosis, miRNAs, noncoding RNAs, AKI, cell death

## Abstract

Cisplatin (*cis*-diamminedichloroplatinum (II), DDP) is an antineoplastic agent widely used in the treatment of solid tumors because of its extensive cytotoxic activity. However, the main limiting side effect of DDP use is nephrotoxicity, a rapid deterioration in kidney function due to toxic chemicals. Several studies have shown that epigenetic processes are involved in DDP-induced nephrotoxicity. Noncoding RNAs (ncRNAs), a class of epigenetic processes, are molecules that regulate gene expression under physiological and pathological conditions. MicroRNAs (miRNAs) are the most characterized class of ncRNAs and are engaged in many cellular processes. In this review, we describe how different miRNAs regulate some pathways leading to cell death by apoptosis, specifically the intrinsic apoptosis pathway. Accordingly, many classes of natural products have been tested for their ability to prevent DDP-induced apoptosis. The study of epigenetic regulation for underlying cell death is still being studied, which will allow new strategies for the diagnosis and therapy of this unwanted disease, which is presented as a side effect of antineoplastic treatment.

## 1. Introduction

Globally, cancer is the second leading cause of death. In 2020, 19.3 million new cases of cancer and almost 10 million people died from this disease [1,2]. In 2022, 1,918,030 new cancer cases and 609,360 cancer deaths are projected to occur in the United States [3]. Cisplatin (*cis*-diamminedichloroplatinum (II), DDP) is an antineoplastic agent widely used in the treatment of solid tumors because of its extensive cytotoxic activity [4,5,6,7,8,9,10]. Despite tremendous advances in oncology, DDP remains a much sought-after chemotherapeutic agent. However, its application has been limited due to several side effects such as nephrotoxicity [11]. Nephrotoxicity is defined as the rapid deterioration in kidney function due to the toxic effect of medications and chemicals [12]. For this to occur, DDP is concentrated and reabsorbed by renal tubular cells (five times more than in the blood), which triggers a rapid decline in renal function [13].

**Figure 1:**
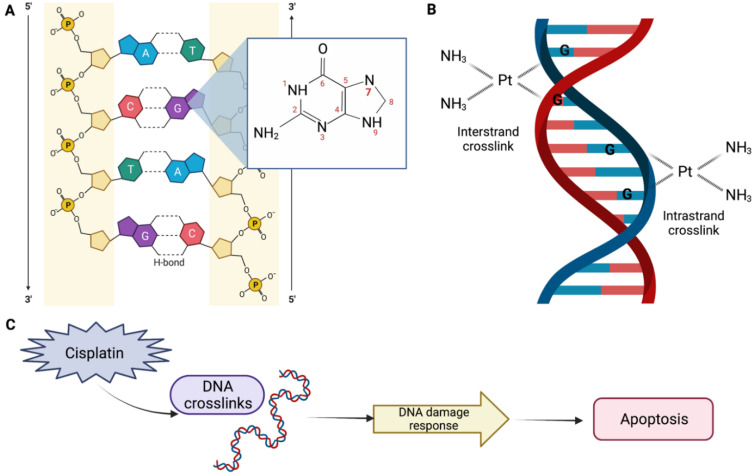
Mechanism of the cisplatin (DDP) action. (**A**) The platinum atom of DDP binds covalently to the N7 position of guanine. (**B**) An interstrand crosslink is formed when DDP binds to two bases from different strands. On the other hand, an intrastrand adduct is formed when DDP binds to two bases of the same strand. (**C**) DDP perturbs genomic DNA, inducing DNA damage response, which may result in apoptosis, DNA repair, and/or cell cycle arrest. Created with BioRender.com.

DDP-induced nephrotoxicity can present in a several ways, but the most common and serious presentation is acute kidney injury (AKI), which occurs in 20–30% of patients. The mechanism of the DDP action is not completely understood. However, it is known that DDP interacts primarily with genomic DNA, specifically the N7 position of guanine bases (Figure 1A), causing inter- and intrastrand DNA crosslinks (Figure 1B), which perturbs DNA synthesis, replication, and transcription, thereby inducing replication stress and DNA damage response. This results in defective DNA strands, which may eventually result in cell apoptosis (Figure 1C) [14,15]. 

The nephrotoxic effect produced by DDP is due to it accumulation in the kidney, mainly in the S3 segment of the proximal tubules, determined by the high density of negatively charged mitochondria in the proximal tubular cells, which attracts positively charged DDP hydrolyzed complexes [16] and contributes critically to sublethal and lethal injury of kidney tubules and the consequent loss of renal function.

Several studies have shown that epigenetic processes are involved in DDP-induced nephrotoxicity in recent years. In 2001, Wu and Morris defined the term “epigenetics” as the study of the changes in gene expression, which occur in organisms with differentiated cells, and the mitotic inheritance of given patterns of gene expression [17]. These modifications result from changes in chromatin structural/activation states without altering the DNA primary nucleotide sequence, triggering the activation of transcription or gene silencing [18]. Noncoding RNAs (ncRNAs), a class of epigenetic processes, are molecules that regulate gene expression under physiological and pathological conditions [19]. MicroRNAs (miRNAs) are small ncRNAs, with an average of 22 bp in length, which have been extensively studied. The latest release of the miRbase database (v22) contains 2654 human mature miRNAs sequences [20], which confirms their importance in gene expression regulation. 

Therefore, understanding the importance of miRNAs during the action of DDP, specifically upon the induction of apoptosis during nephrotoxicity, is of great scientific interest.

## 2. Materials and Methods

### 2.1. Search Strategy

A comprehensive search in Google Scholar, Scopus, Pubmed, and Web of Knowledge databases was carried out, to identify studies published from 1st January 1966 until May 2022, concerning the contribution of microRNAs in DDP-induced apoptosis related to nephrotoxicity. Keyword combinations were used using the following words: cisplatin, AKI, nephrotoxicity, renal cells, microRNA, miR, apoptosis, cell death, and natural products.

### 2.2. Inclusion and Exclusion Criteria

Original articles fulfilling the following search criteria were selected: (1) related to apoptosis, (2) using DDP as the nephrotoxic agent, (3) written in English, (4) fully accessible for the authors (through journal subscriptions, request to authors, open access, among others), and (5) in peer-reviewed journals. Review articles and studies where the primary outcome was DDP resistance or sensitization in cancer were excluded. In addition, all those papers that did not meet the five inclusion criteria previously described were excluded from this study.

## 3. Intrinsic Apoptotic Pathway during DDP-Induced Nephrotoxicity

**Figure 2:**
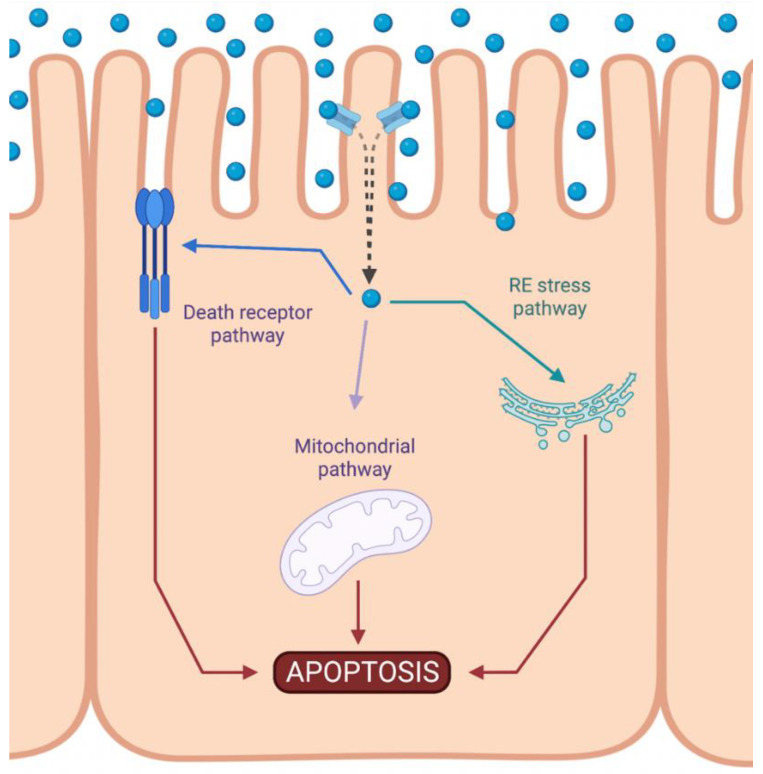
Activation of apoptotic pathways during cisplatin-induced nephrotoxicity. Cisplatin (blue circle) can activate both mitochondrial (purple) and death receptor (blue) pathways of apoptosis. Likewise, endoplasmic reticulum (ER) stress may also be induced (green). Created with BioRender.com.

Cisplatin-induced nephrotoxicity is an adverse side effect of this antineoplastic drug therapy and involves several types of cellular death, such as the necrosis (also called uncontrolled cell death) [21,22,23], apoptosis (programmed cell death) [24,25,26,27,28,29], and necroptosis (regulated inflammatory cell death) [30,31] of renal cells. Concerning programmed apoptosis cell death, the three pathways that have been described are called intrinsic, extrinsic, and endoplasmic reticulum stress (Figure 2). In this review, we focus specifically on the mitochondrial apoptosis pathway.

The intrinsic pathway refers to a primarily mitochondrial-mediated apoptotic pathway that results in cell damage. The intrinsic mitochondrial pathway is the main apoptotic pathway prompted during DDP-induced nephrotoxicity. In this pathway, cellular stress leads to the activation of the proapoptotic B-cell lymphoma 2 (Bcl-2) proteins, Bcl-2-associated X protein (Bax), and Bcl-2 homologous antagonist/killer (Bak) [32], and the reduction of antiapoptotic proteins such as Bcl-2, Bcl-extra-large (Bcl-XL), and myeloid cell leukemia 1 (Mcl-1) [33,34]. This triggers mitochondrial outer-membrane permeabilization (MOMP), releasing apoptotic factors, such as apoptosis-inducing factor (AIF) [35], cytochrome c [36], endonuclease G [37], HtrA2/Omi [38], Smac/DIABLO [39], and others. Chipuk et al. also showed that p53 directly activated Bax and triggered apoptosis [40].

Cytochrome c is a crucial mediator of the mitochondrial pathway. Once in the cytosol, cytochrome c induces a dATP-mediate oligomerization of apoptotic protease-activating factor-1 (Apaf-1) in a 2:1 ratio. This complex then recruits the initiator caspase of this pathway, procaspase-9, and induces its autoactivation [41]. Finally, caspase-9, in turn, activates downstream caspases, such as caspase-3, and initiates the process of caspase-dependent apoptosis [42]. In normal conditions, the second mitochondria-derived activator of caspase (Smac), a protein located in the mitochondria, however, is released into the cytosol when cells undergo apoptosis [43]. Some studies have shown that inhibitors of apoptosis proteins (IAPs), such as XIAP, cIAP1, and cIAP2, are group proteins that negatively regulate both caspases and cell death [44]. Thus, Smac is able to promote caspase-9 activation by binding to IAPs and removing their inhibitory activity [43].

The involvement of the intrinsic pathway of apoptosis in DDP-induced nephrotoxicity has been reported. On in vitro cultured cells, DDP treatment led to a reduction of proapoptotic Bcl-2 protein expression, which was accompanied with an increase of antiapoptotic protein Bax and Bak [33,34,36,45,46,47,48], leading to apoptosis. In the same way, in the rodent model, a reduced Bcl-2 protein expression was also observed after a single dose of DDP [45]. After DDP treatment, the translocation of endogenous Bax from the cytosolic to the membrane fractions was observed and, subsequently, the release of cytochrome c. In addition, using adult Wistar rats, a single dose of DDP triggered severe kidney tissue damage, accompanied by an increase in cytochrome c activity [49]. 

Endonuclease G has also been observed to be induced during DDP injury in mice [50]. Furthermore, using primary mouse proximal tubule cells, Cilenti et al. showed that the level of Omi protein was also upregulated after DDP treatment, and this upregulation was followed by the release of Omi from mitochondria to the cytoplasm, and the subsequent degradation of XIAP [51]. Cisplatin also increases the expression of Apaf-1 [52] and then activates caspase-9/-3 [53], leading to cell death.

## 4. Involvement of microRNAs upon Intrinsic Apoptosis Pathway during DDP-Induced Nephrotoxicity

**Figure 3:**
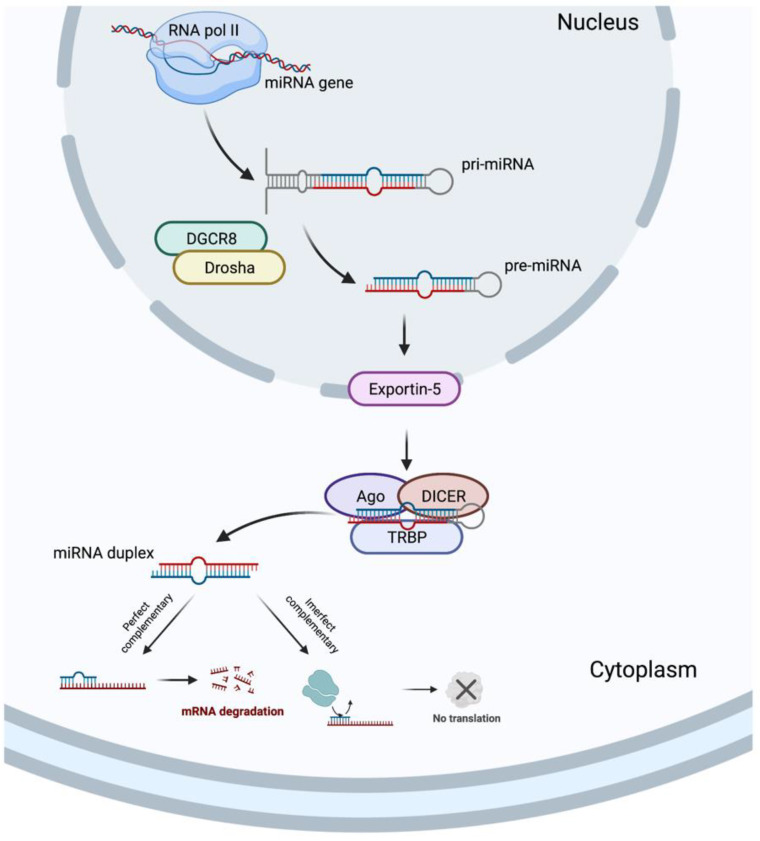
MicroRNA biogenesis. Created with BioRender.com.

In 1993, two different studies, by Lee et al. [54] and Wightman et al. [55], discovered the miRNAs, which revolutionized the field of molecular biology. MicroRNAs are a class of ncRNAs of approximately 22 bp in length that recognize target sites, most commonly found in the 3′-untranslated regions (3′-UTRs) of mRNAs, through imperfect base-pairing, with one or more mismatches in sequence complementarity [56]. Briefly, the miRNAs biogenesis is started when a pri-miRNA is transcribed from its miRNA gene and then recognized and cleaved into pre-miRNA by RNA binding protein DiGeorge Syndrome Critical Region 8 (DGCR8) and a ribonuclease III enzyme, Drosha [57], resulting in the formation of a pre-miRNA, which is exported to the cytoplasm by an Exportin-5 and then processed by the RNase III endonuclease, DICER, in collaboration with the transactivation response RNA binding protein (TRBP) and Argonaute (Ago), which remove the terminal loop, resulting in a mature miRNA duplex [58]. Both 5p or 3p strands originated from the 5′ or 3′ end of the pre-miRNA harping, respectively, are derived from the mature miRNA duplex. Thus, the final result is the generation of a miRNA, which can negatively regulate gene targets at the post-transcriptional level by perfect complementarity of their “seed” region to the 3′-UTR of its target mRNA, inducing their degradation, or by an imperfect complementarity, resulting in translational repression [59] (Figure 3).

In the following, recent progress of miRNA-targeted therapeutics is described, and potential applications in the treatment of DDP-induced nephrotoxicity are discussed. Related to the intrinsic pathway, several miRNAs have been described to regulate apoptosis-related signaling molecules, which are summarized in Table 1.

Using a gene microarray analysis, Zhu et al. [60] demonstrated that renal tissues from the acute kidney injury (AKI) rats caused an upregulation in 36 miRNAs and downregulation in 8 miRNAs. They selected miR-146b for its pronounced changes and verified the increase of miR-146b in the kidneys of AKI rats and in NRK-52E cells treated with DDP. Moreover, transfection with the miR-146b inhibitor reduced the apoptotic rate of NRK-52E cells by directly targeting ErbB4. In DDP-induced apoptosis, restoring the in vitro inhibition of miR-377 expression in tubular epithelial cells after DDP-induced kidney injury in mice was restored by using mesenchymal stem cells (MSCs), since they promoted an increase in the expression of cytoprotective genes, such as Bcl-2 [48]. Mesenchymal stem cells are stromal cells that can self-renew and that also exhibit a multilineage differentiation [61]; they have been verified to be a safe and effective delivery vehicle for therapeutic miRNA treatment [62]. Another bioinformatic research study also demonstrated that miR-1184 was downregulated in AKI. To obtain this, exosomal-miR-1184-derived MSCs alleviated DDP-induced HK-2 cell injury, observed by the downregulation of the expression levels of Bax and cleaved caspase-3 and the upregulation of the protein expression level of Bcl-2 [63]. In the same way, bone marrow MSCs also downregulated miR-107 expression induced after the DDP stimulus, which increased the level of RPS19, and finally inhibited DDP-induced apoptosis by reducing Bcl-2 protein expression [64]. Another study demonstrated that the treatment with urinary exosomes from premature infants alleviated DDP-induced AKI in mice and inhibited the apoptosis of HK-2 cells by reducing the expression of Bax and increasing the expression of Bcl-2 via miR-30a-5p and targeting MAPK8 [45].

The ability of DDP to induce cell death requires the sequential activation of the p53/ROS/p38a MAPK cascade [77]. On this basis, a microarray analysis identified 47 differentially expressed miRNAs during DDP-cytotoxicity of HK-2 cells. Moreover, a pathway analysis indicated that the top upregulated pathways included the MAPK and p53 signaling pathways. A further network analysis showed that the MAPK signaling pathway and apoptosis were identified as core pathways and miR-9-3p and miR-371b-5p as the most critical miRNAs during DDP-induced cytotoxicity [65]. In rat renal proximal tubular cells, DDP-induced miR-449 upregulation was able to inhibit SIRT1 expression, which further elevated acetylated p53 and BAX levels, leading to the p53/BAX signaling of the intrinsic apoptosis pathway. Nevertheless, inhibiting miR-449 expression in DDP-treated cells suppressed cell apoptosis [33]. After sequencing and a subsequent qRT-PCR, Han et al. [69] revealed that miR-132-3p was upregulated after DDP treatment in mouse and HK-2 cells. Moreover, they also found that apoptosis was suppressed by inhibiting the miR-132-3p expression in DDP-stimulated HK-2 cells, and this suppression was blocked by miR-132-3p mimics and exacerbated DDP-induced AKI by negatively regulating SIRT1 and activating the NF-κB signaling pathway. Yang et al. [75] also demonstrated that p53 was upregulated in DDP-induced AKI mice. They also observed that pifithrin-α inhibited the p53 expression and attenuated renal injury in vivo and cell apoptosis in vitro in mice and HK-2 cells, respectively. In addition, they identified that p53 regulated miR-199a-3p expression and blocking miR-199a-3p reduced DDP-induced apoptosis in HK-2 cells. In addition, the treatment of NRK-52E cells with this antineoplastic demonstrated that DDP facilitated the association of FOXO3 and p53 and was parallel with the accumulation of Bax. Furthermore, the overexpression of miR-122 diminished p53 and Bax levels in NRK-52E cells treated with DDP, while the overexpression of miR-34a promoted both the basal and the inducible expression of p53 and Bax [67].

p53 mediates cisplatin-induced apoptosis in renal proximal tubular cells, and p53 can activate caspase-3 [78]. Related to this, Li et al. [76] described that HRPTEp cells treated with DDP for 48 h showed that DDP led to significantly upregulated miR-449a. In the same way, the overexpression of miR-449a led to an increased apoptotic rate of HRPTEpCs after DDP insult, while antagomir-449a reversed it. Another study demonstrated that HK-2 cells stimulated with DDP showed that the downregulation of miR-205-5p promoted cell apoptosis, observed by an enhanced caspase-3 activity and apoptosis rate of in vitro cultured cells. However, enhancing miR-205-5p expression suppressed apoptotic rate [72]. The levels of miR-144-5p were also downregulated in DDP-stimulated HK-2 cells, and the expression levels of apoptosis-related proteins showed that enhancing miR-144-5p expression was able to increase the expression levels of caspase-3/-9, and Bax, and to decrease the expression levels of Bcl-2, by regulating PKM2 expression [71]. Opposite results were observed by Zhang et al. [70], since they demonstrated that remote ischemic preconditioning, a strategy to induce resistance in a target organ, exerted a protective effect on DDP-induced AKI in mice by reversing the downregulation of miR-144 and the dysregulation of caspase-3, Bax, and Bcl-2 expression in renal tissues of DDP-induced AKI in mice and NRK-52 cells. A similar study also showed that an enhanced miR-182-5p expression could reduce renal epithelial Bcl-2 levels and promote Bax and cleaved caspase-3 after in vitro DDP insult, and inhibiting its expression attenuated the damage of DDP in HK-2 cells [74]. Zhu et al. [34] found that miR-181a expression downregulated after DDP-induced apoptosis. They also found that Bcl-2 was upregulated and Bax was downregulated after the transient transfection of the miR-181a inhibitor in HK-2 cells, suggesting that miR-181a was directly involved in the apoptotic process. Using qRT-PCR analysis, Zhang et al. [73] observed a downregulation of miR-205 in HK-2 cells treated with DDP. The transient overexpression of miR-205 using mimics demonstrated that HK-2 cells were more resistant to DDP-induced apoptosis, by modulating CMTM4 protein expression. In another study, qPCR was performed to evaluate the miR-125b expression in in vitro and in vivo DDP-induced damage, and the results showed the upregulation of miR-125b after DDP injection and in cultured tubular epithelial cells treated with DDP. Moreover, DDP-induced apoptosis was decreased by using a miR-125b inhibitor, as shown by the TUNEL assay and the reduced Bax expression [68]. Another study demonstrated that DDP notably increased miR-31 expression and apoptosis-associated proteins (caspase-3 and Bax), while decreasing the antiapoptotic factor, Bcl-2, in kidney samples [66].

Thus, it is evident that many miRNAs exert a fundamental role in activating or inhibiting critical molecules in the progression of the mitochondrial pathway of apoptosis.

## 5. Potential Utility of Natural Products in DDP-Induced Apoptosis

Cisplatin is an effective chemotherapeutic drug whose clinical use and efficacy are limited by its nephrotoxicity, which affects mainly the renal tubular cells. It accumulates in proximal and distal epithelial tubule cells and causes cell death. Consequently, various classes of natural products have been tested for their capacity to prevent DDP-induced nephrotoxicity. Furthermore, natural products also overcome resistance, sensitizing cancer cells to DDP [79].

Currently, there is no effective drug to avoid or treat DDP-induced nephrotoxicity. As a result, multiple drugs from natural products have been developed to protect against DDP-induced side effects. All the following natural products we describe have been shown to reduce, alleviate, or mitigate DDP-induced nephrotoxicity by regulating DDP-induced mouse tubular epithelial cells apoptosis by inhibiting the expression of p53, Bax, and cleaved caspase-3/-9 and activating the expression of Bcl-2 both in in vitro cultured HEK-293, HK-2 and/or LLC-PK1 cells and in in vivo DDP-induced AKI mice and/or rats. 

Alkaloids are a class of natural compound. Alkaloids represent a vast group of naturally occurring compounds which contain at least one nitrogen atom (amino or amido in some cases). Some alkaloids shown to prevent DDP-induced apoptosis include the berberine [80,81], betaine [49,82], boldine [83], and ligustrazine [84,85].

Flavonoids are a class of polyphenolic secondary metabolites found in plants. Several studies have shown that the use of certain flavonoids prevent DDP-induced apoptosis, most notably the use of astilbin [86], cyanidin [87], epicatechin gallate [88], farrerol [89], galangin [90,91], hespertin [92], icariin [93,94], isoliquiritin [95], isoorientin [96], isoquercitrin [97], luteolin [98,99], morin [100], naringin [101], puerarin [66], quercetin [102,103], rutin [104], scutellarin [105], silybin [106], silymarin [107,108], and wogonin [109,110].

Another class of natural product consists of phenolic compounds. They are secondary metabolites produced in the shikimic acid of plants, which contain benzene rings, with one or more hydroxyl substituents. Phenolic compounds with research related to this side effect include curcumin [111,112,113,114,115], ellagic acid [116], epigallocatechin gallate [117,118], ferulic acid [115], honokiol [119], hydroxytyrosol [120], oleuropein [121], punicalagin [122], rosmarinic acid [123], sinapic acid [124], and zingerone [125].

Likewise, terpenoids are a large and diverse group of lipids resulting from five-carbon isoprene units assembled in thousands of combinations and are isolated from plants and microbial sources. The following terpenoids have shown potential in some studies: anemoside B4 [126], carnosic acid [127], carvacrol [128,129], dioscin [130], germacrone [131], ginsenoside 20 (S)-RG3/Re/RG5/Rk1/Rh2 [132,133,134,135,136], linalool [137], *Panax quinquefolius* saponins [138], *Panax notoginseng* saponins [139,140], platycodin D [141,142], pseudoginsengenin DQ [53], red ginseng [143], saikosaponin D [144], and *Terminalia arjuna* triterpenoid saponins [145].

However, only a few studies have shown the use of antioxidants in epigenetic regulation, specifically on miRNAs, during DDP-induced apoptosis. For example, betanin, a natural red glycoside food dye obtained from beets, has been shown to reduce organ damage induced by DDP by reducing miRNA-34a expression and enhancing the SIRT1/PGC-α pathway [146]. Moreover, puerarin, another natural flavonoid extracted from the Chinese medical herb *Radix puerariae*, alleviated DDP-induced AKI by suppressing miR-31 expression, enhancing Numb activation, thereby inhibiting the Notch signaling pathway [66]. Finally, dioscin, a steroid saponin commonly found in various herbs, protected against DDP-induced injury to NRK-52E and HK-2 cells by decreasing miR-34a expression, which was accompanied by increased levels of SIRT1, and thus, cell damage [130].

Therefore, natural products could be developed as a new candidate to alleviate DDP-induced cell injury. In addition, they could be used as an ncRNA-based therapy to counteract apoptosis and other pathways induced during nephrotoxicity. 

## 6. Conclusions

Nephrotoxicity is the main side effect of DDP treatment. Therefore, understanding the epigenetic mechanisms underlying the side effect of nephrotoxicity may contribute to implementing therapeutic strategies to alleviate this side effect of chemotherapeutic treatment. This review revealed the complexity of the interactions between miRNAs with their respective targets, how this contributed to the induction of the intrinsic apoptosis pathway, and some natural strategies for overcoming this side effect. Thus, it provided a starting point for developing ncRNA-based therapies to accelerate the resolution of apoptosis induced during nephrotoxicity, thus improving the quality of life of patients treated with DPP. 

## Figures and Tables

**Table 1:** MicroRNAs (miRNAs) involved in cisplatin (DDP)-induced apoptosis-related signaling molecules.

ID	Regulation in DDP-Induced Apoptosis	Main Findings on Apoptosis-Related Signaling Molecules	Reference
miR-9-3p	Up	After a microarray assay, a network analysis showed that miR-9-3p and miR-371b-5p were the most critical miRNAs during DDP-induced cytotoxicity.	[65]
miR-30a-5p	Down	Urinary exosomes derived from premature infants alleviated DDP-induced apoptosis of HK-2 by augmenting Bcl-2 expression and reducing caspase-3 activity due to an upregulation of miR-30a-5p, which led to the reduced MAPK8 protein expression.	[45]
miR-31	Up	Puerarin alleviated DDP-induced AKI by suppressing miR-31 expression, enhancing Numb activation, and inhibiting the Notch signaling pathway.	[66]
miR-34a	Up	miR-34a was elevated in the kidney after DDP treatment. The increase in miR-34a activated FOXO3 by suppressing SIRT1, favoring a shift in tubular cell viability toward cell cycle arrest or apoptosis.	[67]
miR-107	Up	Mesenchymal stromal cells downregulated miR-107 expression induced after DDP stimulus, inhibiting DDP-induced apoptosis by reducing Bcl-2 protein expression.	[64]
miR-122	Down	miR-122 was decreased in the kidney after DDP treatment. The reduction in miR-122 activated FOXO3, favoring a shift in tubular cell viability toward cell cycle arrest or apoptosis.	[67]
miR-125b	Up	Inhibiting miR-125b expression exerted mitochondrial and renal protection in DDP-damaged HK-2 cells.	[68]
miR-132-3p	Up	Inhibition of miR-132-3p protected against DDP-induced AKI via the SIRT1/NF-κB pathway.	[69]
miR-144	Down	Remote ischemic preconditioning alleviated the renal functional and histopathological damage of DDP-induced AKI in mice and in NRK-52 cells by the upregulation of miR-144 and the downregulation of its target PTEN.	[70]
miR-144-5p	Up	Overexpression of miR-144-5p increased expression of caspase-3/-9, and Bax, and also decreased levels of Bcl-2 in DDP-stimulated HK-2 cells.	[71]
miR-146b	Up	Inhibition of miR-146b expression reduced the apoptotic rate of NRK-52E cells by directly targeting ErbB4.	[60]
miR-205-5p	Down	Enhancing miR-205-5p expression suppressed caspase-3 activity and apoptosis rate of in vitro cultured HK-2 cells.	[72]
	Down	Overexpression of miR-205 in HK-2 cells demonstrated that they were more resistant to DDP-induced apoptosis by directly targeting CMTM4.	[73]
miR-181a	Up	Promoted apoptosis by decreasing Bcl-2 and enhancing Bax expression of HK-2 cells.	[34]
miR-182-5p	Up	Enhanced miR-182-5p, demonstrated to reduce renal apoptosis of HK-2 cells by reducing Bcl-2 levels and promoting Bax and cleaved caspase-3 after in vitro DDP insult.	[74]
miR-199a-3p	Up	Inhibiting the p53 expression, by using pifithrin-α, attenuated renal injury and cell apoptosis in mice and HK-2 cells, respectively. Blocking miR-199a-3p reduced DDP-induced apoptosis in HK-2 cells.	[75]
miR-371b-5p	Up	After a microarray assay, a network analysis showed that miR-9-3p and miR-371b-5p were the most critical miRNAs during DDP-induced cytotoxicity.	[65]
miR-377-3p	Up	Mesenchymal stromal cells restored tissue function after DDP-induced kidney injury in mice, by promoting the increase in the expression of cytoprotective genes, such as Bcl-2, due to the inhibition of miR-377 in tubular epithelial cells.	[48]
miR-449a	Up	Overexpression of miR-449a led to the increased apoptotic rate of HRPTEpCs after DDP treatment, while antagomiR-449a reversed this effect.	[76]
miR-449	Up	Enhanced cell apoptosis of NRK-52E cells, observed by decreased SIRT1 and increased phosphorylated-p53 and BAX expression.	[33]
miR-1184	Down	Enhanced miR-1184 expression by using miR-1184 agomir, downregulated Bax, and upregulated Bcl-2 protein expression, thus reducing DDP-induced HK-2 cell injury.	[63]

## Data Availability

Not applicable.
